# Crystal structure of human interferon-γ receptor 2 reveals the structural basis for receptor specificity

**DOI:** 10.1107/S2059798316012237

**Published:** 2016-08-18

**Authors:** Pavel Mikulecký, Jirí Zahradník, Petr Kolenko, Jiří Černý, Tatsiana Charnavets, Lucie Kolářová, Iva Nečasová, Phuong Ngoc Pham, Bohdan Schneider

**Affiliations:** a Institute of Biotechnology CAS, BIOCEV, Prumyslova 595, 252 50 Vestec, Czech Republic

**Keywords:** interferon-γ receptor 2, fibronectin type III domain, class 2 cytokine receptors

## Abstract

Interferon-γ receptor 2 is a cell-surface receptor that is required for interferon-γ signalling and therefore plays a critical immunoregulatory role in innate and adaptive immunity against viral and also bacterial and protozoal infections. A crystal structure of the extracellular part of human interferon-γ receptor 2 (IFNγR2) was solved by molecular replacement at 1.8 Å resolution. Similar to other class 2 receptors, IFNγR2 has two fibronectin type III domains. The characteristic structural features of IFNγR2 are concentrated in its N-terminal domain: an extensive π–cation motif of stacked residues KWRWRH, a NAG–W–NAG sandwich (where NAG stands for *N*-acetyl-d-glucosamine) and finally a helix formed by residues 78–85, which is unique among class 2 receptors. Mass spectrometry and mutational analyses showed the importance of N-linked glycosylation to the stability of the protein and confirmed the presence of two disulfide bonds. Structure-based bioinformatic analysis revealed independent evolutionary behaviour of both receptor domains and, together with multiple sequence alignment, identified putative binding sites for interferon-γ and receptor 1, the ligands of IFNγR2.

## Abstract 2

The structure of the extracellular portion of interferon-γ receptor 2, a member of the class II cytokine receptors, has been solved. Bioinformatic analysis revealed independent evolutionary behaviour of both receptor domains and identified a putative binding site for interferon-γ.

## 1. Introduction   

Interferon-γ receptor 2 is a cell-surface receptor that represents a crucial molecule in the interferon-γ (IFNγ) signalization pathway, influencing innate and adaptive immunity against pathogens and tumours (Schoenborn & Wilson, 2007 ▸; Lin & Young, 2013 ▸). The signalling cascade is initiated by the binding of IFNγ to its high-affinity cell surface receptor 1, forming a binary complex with a structure that has already been determined [PDB entries 1fg9 (Thiel *et al.*, 2000 ▸) and 1fyh (Landar *et al.*, 2000 ▸)]. However, to activate this binary complex and activate the JAK/STAT signalization pathway (Jung *et al.*, 1987 ▸; Cook *et al.*, 1994 ▸; Hemmi *et al.*, 1994 ▸), IFNγ receptor 2 must participate in a ternary complex created by a homodimer of IFNγ, two molecules of receptor 1 and molecule(s) of receptor 2. To date, detailed structural and biophysical characterization of IFNγ receptor 2, the key molecule for proper IFNγ signalization, is lacking.

From its sequence similarity, IFNγ receptor 2 (also known as IFNγ receptor β chain or accessory factor 1, AF-1), has been classified as a member of the class 2 receptor family. This large group of cytokine receptors includes IFNγ receptor 1, receptors of interferon-α and interferon-β, receptors of interleukin-10 and interleukin-20, and receptors of other interleukins belonging to the IL-10 family (Langer *et al.*, 2004 ▸). The mature IFNγ receptor 2 protein comprises of 310 amino acids and has a predicted molecular mass of 35 kDa. It consists of a relatively short 69-amino-acid intracellular domain, a 21-amino-acid transmembrane domain and a 220-amino-acid extracellular domain that is structured into two fibronectin type III domains. The extracellular domain contains five cysteine residues and six potential N-linked glycosylation sites. Such extensive glycosylation contributes to a significant size heterogeneity, which is observed even when the receptor molecule is isolated from the same cell type; the molecular weight of mature receptor 2 of human interferon-γ ranges from 61 to 67 kDa (Bach *et al.*, 1995 ▸).

Despite its biological significance, three-dimensional structural data on IFNγ receptor 2 are lacking. Here, we report a 1.8 Å resolution crystal structure of the extracellular portion of IFNγ receptor 2 (hereafter called IFNγR2); the structure has been deposited in the Protein Data Bank as entry 5eh1. The structure and sequence of IFNγR2 are discussed in the context of the structures and sequences of the related class 2 cytokine receptors, with emphasis on the sequentially closest receptors of interleukins from the IL-10 family. Structure-based and sequence-based alignments suggested regions securing binding specificity of these receptors for their cytokine ligands.

## 2. Materials and methods   

### 2.1. Cloning, expression and purification of IFNγR2   

The gene encoding the extracellular part of IFNγR2 (residues 28–247 of UniProt entry P38484) was cloned into a *Drosophila* pMT-BiP-V5-His_A vector using BglII and AgeI restriction enzymes in frame with an N-terminal BiP signal peptide and a C-terminal 6×His tag. This expression vector was co-transfected into insect Schneider S2 cells along with the pCoBlast selection plasmid using Effectene Transfection Reagent according to the manufacturer’s instructions. Blasticidin-resistant S2 cells were selected by growing the cells in HyClone SFX Medium supplemented with 10% FBS and 25 µg ml^−1^ blasticidin S. Large-scale protein expression was achieved after expansion and substitution into HyClone SFX serum-free medium, and protein expression was induced by the addition of 0.75 m*M* CuSO_4_ for 6 d (the cell concentration was approximately 35 million per millilitre) and 1.5 m*M* CuSO_4_ for a further 2 d until the percentage of living cells did not decrease below 95%. After expression, the cells were discarded by centrifugation and the medium containing secreted glycosylated IFNγR2 protein was supplemented with the following additives at the following final concentrations: 5 m*M* CaCl_2_, 1 m*M* NiSO_4_, 250 m*M* NaCl and 50 m*M* Tris–HCl pH 8. The protein was purified on an IMAC HP column charged with NiSO_4_ and equilibrated with EQ buffer (50 m*M* Tris–HCl pH 8, 500 m*M* NaCl). The column was washed with W buffer (50 m*M* Tris–HCl pH 8, 500 m*M* NaCl, 20 m*M* imidazole pH 8) and the protein was eluted with EL buffer (50 m*M* Tris–HCl pH 8, 500 m*M* NaCl, 250 m*M* imidazole pH 8). It was further purified to homogeneity by size-exclusion chromatography at room temperature on a HiLoad 16/600 Superdex 200 pg column (GE Healthcare) equilibrated with HN buffer (10 m*M* HEPES pH 7.5, 100 m*M* NaCl). Samples were analyzed by 12% SDS–PAGE.

IFNγR2 was produced in insect cells as a secreted protein bearing oligosaccharide moieties of approximately 10 kDa according to SDS–PAGE analysis. Deglycosylation by peptide:N-glycosidase F (PNGase F) or endoglycosidase H (Endo H) with a C-terminal *Strep*-tag (§S1, Supporting Information) was performed after purification of IFNγR2 on an IMAC column during dialysis against TN buffer (50 m*M* Tris buffer pH 8, 150 m*M* NaCl) or HN buffer, respectively. Endoglycosidases were removed on a *Strep*-Tactin column and the nonbound fraction containing IFNγR2 was further purified by size-exclusion chromatography in HN buffer.

The single IFNγR2 variants (N110Q, N137Q and N231Q, respectively) were introduced using the QuikChange II Site-Directed Mutagenesis Kit (Agilent Technologies). Primers are listed in Supplementary Table S1. The fully mutated IFNγR2 variant bearing N56Q, N110Q, N137Q and N231Q mutations was obtained as a GeneArt Strings DNA Fragment and was cloned with the same protocol as the wild type. The expression and purification of all IFNγR2 variants were performed in the same way as described above.

### 2.2. Biophysical measurements   

Circular-dichroism (CD) spectra were recorded using a Chirascan-plus spectrometer (Applied Photophysics) in steps of 1 nm over the wavelength range 185–260 nm. Samples diluted with water to a concentration of 0.2 mg ml^−1^ were placed into the holder in a 0.05 cm path-length quartz cell and individual spectra were recorded at a temperature of 23°C. The CD signal was expressed as the ellipticity and the resulting spectra were buffer-subtracted. To analyze the ratio of secondary structures, we used the *CDNN* program (Böhm *et al.*, 1992 ▸) provided with the Chirascan CD spectrometer. CD melting measurements were performed using samples diluted with water to a protein concentration of 0.5 mg ml^−1^. A 10 mm path-length quartz cell was placed into the thermostated holder and sample absorption was recorded at 280 nm in 1°C increments at a rate of 0.5°C min^−1^ over the temperature range 20–85°C with an averaging time of 12 s. Melting curves were normalized to relative values between 0.0 and 1.0 to visually magnify differences between the melting profiles, and the melting temperature (*T*_m_) was estimated from the first derivative of the melting curves.

### 2.3. Glycosylation analysis and disulfide-bond determination   

IFNγR2 glycosylation sites were determined by MALDI-MS analysis preceded by protein digestion as described previously (Plíhal *et al.*, 2004 ▸). Disulfide bonds in IFNγR2 were determined by SDS–PAGE and subsequent identification by mass spectrometry (MS) in analogy to the previously described procedure (Pompach *et al.*, 2009 ▸). 20 µg of sample in nonreducing conditions was loaded onto a 4–12% gradient gel (Life Technologies) in the presence of 200 µ*M* cystamine. Bands corresponding to highly glycosylated IFNγR2 were excised and subjected to in-gel deglycosylation and proteolysis. Deglycosylation using Endo H (New England Biolabs) was carried out for 4 h at 37°C and the resulting partly deglycosylated sample was digested with trypsin (sequencing grade, Promega) for 12 h at 37°C at a protein:enzyme ratio of 30:1(*w*/*w*). After digestion, the tryptic peptide mixture was desalted on a peptide MicroTrap column (Michrom Bio­resources) and separated on a reversed-phase C18 column (Acclaim PepMap 100, 5 µm, 0.1 × 20 mm; Thermo Scientific). The mobile phases consisted of 0.1% formic acid in 2% aceto­nitrile (solvent *A*) and 0.1% formic acid in 98% aceto­nitrile (solvent *B*). Peptides were eluted under the following gradient conditions: 2–45% solvent *B* in 40 min, 45–95% solvent *B* in 5 min. The flow rate was 0.5 µl min^−1^ and the column was directly connected to the mass spectrometer. Mass spectra were acquired on a solariX XR FTMS instrument equipped with a 12 T superconducting magnet (Bruker Daltonics). For the identification of disulfide bonds, we used the *Links* algorithm, previously described as *ASAP* (*Automated Spectrum Assignment Program*; Schilling *et al.*, 2003 ▸). To generate deconvoluted spectra and export the *m*/*z* values, we used a script utilizing the *SNAP* 2.0 algorithm of the *DataAnalysis* 4.2 software suite (Bruker Daltonics).

### 2.4. Crystallization and diffraction data collection   

Crystals of Endo H-deglycosylated IFNγR2 receptor were grown using the sitting-drop vapour-diffusion method in 96-3 three-well Intelli-Plate trays (Art Robbins Instruments). The reservoir solution consisted of 0.1 *M* MES pH 5.0, 10% PEG 6000 (final pH 6.0): condition No. 61 of The JCSG Core I Suite (Qiagen). Drops consisting of 0.2 µl protein sample (15 mg ml^−1^ protein in HN buffer) and 0.2 µl reservoir solution were prepared with a Gryphon liquid-pipette robot (Dunn Labortechnik) and were equilibrated against 100 µl reservoir solution. Crystals appeared after 30 d of incubation at 291 K. Crystals were mounted in Round LithoLoops (Molecular Dimensions) and flash-cooled in liquid nitrogen after cryoprotection in 20%(*v*/*v*) glycerol. X-ray diffraction data were collected at 100 K on beamline MX 14.1 of the BESSY II synchrotron-radiation source at the Helmholtz-Zentrum Berlin (HZB). A native data set was collected at a wavelength of 0.918 Å.

### 2.5. Data processing, structure determination and refinement   

The diffraction and refinement statistics are summarized in Table 1 ▸. Diffraction data were processed and scaled using the *XDS* program package (Kabsch, 2010 ▸). The structure was solved with *BALBES* (Long *et al.*, 2008 ▸), but the structure model needed significant manual remodelling. Only the C-terminal domain of IFNγR2 was found and the initial *R* factors were about 0.49 and 0.52 for *R*_work_ and *R*_free_, respectively. Residues missing from the initial model were built in with significant help from *ARP*/*wARP* (Langer *et al.*, 2008 ▸); manual corrections and building were performed using *Coot* (Emsley & Cowtan, 2004 ▸). Refinement was then carried out with *REFMAC*5 (Murshudov *et al.*, 2011 ▸) and the structure was validated by *MolProbity* (Chen *et al.*, 2010 ▸). The coordinates and structure factors have been deposited in the PDB with accession code 5eh1.

### 2.6. Sequence and structural bioinformatics   

The UniProt database was searched with the *BLAST* tool (Camacho *et al.*, 2009 ▸) using the sequence of the extracellular part of IFNγR2 as the query sequence. The automated result was manually reviewed to select 90 sequences from different species. These sequences were used to calculate a multiple sequence alignment with *Clustal Omega* (Sievers *et al.*, 2011 ▸) as implemented in *UGENE* (Okonechnikov *et al.*, 2012 ▸). The *ConSurf* server (Glaser *et al.*, 2003 ▸) was used to estimate the evolutionary conservation of amino-acid positions in the protein structures. The calculations were based on the crystal structure of IFNγR2 (PDB entry 5eh1) and the alignment prepared by *Clustal Omega*. Structural comparison was prepared by *MatchMaker* as implemented in the *UCSF Chimera* software (Pettersen *et al.*, 2004 ▸).

Root-mean-square deviation (r.m.s.d.) values between the N- and C-terminal domains were calculated using *VMD* (Humphrey *et al.*, 1996 ▸). The backbone atoms of 34 sequentially conserved residues in each domain (listed in Supplementary Table S2) were used for the structure superposition of all possible pairs of N- and C-terminal domains of the 12 available crystal structures of class 2 cytokine receptors. The *VMD* commands measure fit and move were used for the structural overlay, followed by measure rmsd operating on the same selection of residues and backbone atoms for the calculation of r.m.s.d. values. The stability of the N-terminal domain of IFNγR2 was estimated by calculating the pairwise interaction energy at the DFT-D level as detailed in Supplementary Fig. S3.

## 3. Results and discussion   

### 3.1. Summary   

**Figure 1:**
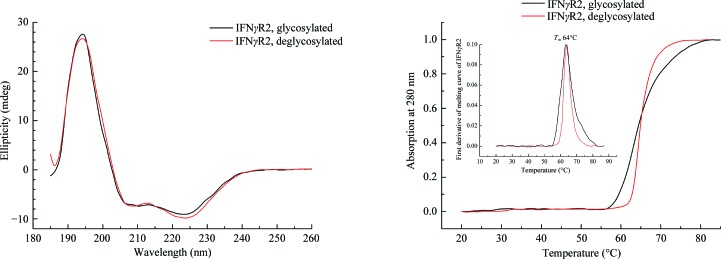
Left: circular-dichroism (CD) spectra of glycosylated and deglycosylated IFNγR2. The CD spectra of both proteins are highly similar, suggesting that the partial removal of the oligosaccharide moieties did not affect the overall structure of IFNγR2. Right: normalized melting curves measured from temperature-dependent CD spectra at 280 nm. The melting temperature was estimated as 64°C for both glycosylated and deglycosylated IFNγR2.

**Figure 2:**
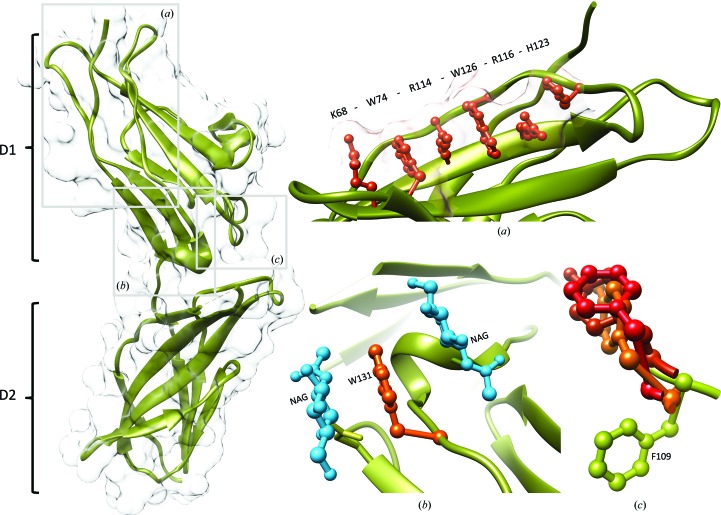
Left: ribbon and surface representations of the IFNγR2 structure. D1 and D2 indicate domains 1 and 2, respectively. Insets: (*a*) residues Lys68, Trp74, Arg114, Trp126, Arg116 and His123 of the D1 domain form a stacking motif on the IFNγR2 surface. (*b*) *N*-Acetyl-d-glucosamines (NAGs; blue) glycosylating Asn110 and Asn137 sandwich Trp131 (orange), reducing its hydrophobic character. (*c*) The superposition of aromatic binding epitopes shows differences between IFNγR2 (Phe109 in green) on one side and promiscuous shared cytokine receptors on the other [in red; Tyr82 of IL10R2 (PDB entry 3lqm; Yoon *et al.*, 2010 ▸), Phe169 of gp130 (PDB entry 1bqu; Bravo *et al.*, 1998 ▸) and Tyr103 of γ_c_ receptor (PDB entry 4gs7; Ring *et al.*, 2012 ▸)]. No corresponding aromatic residue is observed in IL20R2 (Logsdon *et al.*, 2012 ▸).

**Figure 3:**
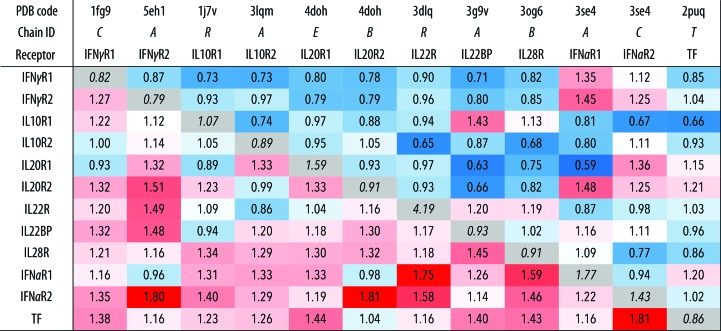
Structural differences between the N-terminal (D1) and C-terminal (D2) domains of 12 class 2 cytokine receptors gauged by the r.m.s.d. values for backbone atoms of their 34 residues. R.m.s.d. values comparing D1 and D2 domains are shown above and below the diagonal, respectively. For instance, comparison between D1 of IL20R2 and IL22BP gives an r.m.s.d. of 0.66 Å; the r.m.s.d. between their D2 domains is 1.30 Å. R.m.s.d. values that are smaller and larger than the off-diagonal average r.m.s.d. value are highlighted in blue and red hues, respectively. The diagonal (in grey) shows the lowest r.m.s.d. values for 34 residues from D1 and D2 within each receptor structure; the r.m.s.d. between D1 and D2 of IL10R2 is 0.89 Å. References to the analyzed structures are as follows: IFNγR1, Thiel *et al.* (2000 ▸); IFNγR2, this work; IL10R1, Josephson *et al.* (2001 ▸); IL10R2, Yoon *et al.* (2010 ▸); IL20R1 and IL20R2, Logsdon *et al.* (2012 ▸); IL22R, Bleicher *et al.* (2008 ▸); IL22BP, de Moura *et al.* (2009 ▸); IL28R, Miknis *et al.* (2010 ▸); IFNαR1 and IFNαR2, Thomas *et al.* (2011 ▸); TF (human tissue factor), Larsen *et al.* (2007 ▸).

IFNγR2 was produced in insect S2 cells, purified, characterized by biophysical techniques (Fig. 1 ▸) and its crystal structure was solved at 1.8 Å resolution. Fig. 2 ▸ highlights some of the structural features of IFNγR2; Figs. 3 ▸ and 4 ▸ provide a comparison of IFNγR2 to the other cytokine receptors from the class 2 family, with the aim of correlating the sequences and structures of these proteins.

### 3.2. Glycosylation and overall fold stability   

The IFNγR2 protein has six potential glycosylation sites (Asn56, Asn110, Asn137, Asn231, Asn85 and Asn219), of which the first four were confirmed by mass-spectrometric analysis as glycosylated in our construct; the crystal structure later revealed that position Asn85 was also glycosylated. The greatest heterogeneity was observed at position Asn137. To remove oligosaccharide moieties from these residues, we used two endoglycosidases, Endo H and PNGase F, but both enzymes left several forms of IFNγR2 with residual glycosyl­ation as observed by a distribution of molecular mass on SDS–PAGE (Supplementary Fig. S1). Mass spectrometry identified α(1–3)-fucose at position Asn231. Because α(1–3)-fucose abolishes the activity of both endoglycosidases, its presence is a likely to be reason for the mass distribution of IFNγR2. Deglycosylation by Endo H caused an approximately 7 kDa shift in molecular weight on SDS–PAGE and, in contrast to deglycosylation by PNGase, did not induce protein oligomerization, as checked by size-exclusion chromatography. Measurements using CD spectroscopy (Fig. 1 ▸) and thermal shift assay (§S1 in Supporting Information and Supplementary Fig. S2) showed no significant difference in melting temperatures between the glycosylated and Endo H-deglycosylated forms of the IFNγR2 protein. Because the CD spectra of these two forms are also virtually identical, we believe that deglycosylation does not influence the secondary structure of IFNγR2. Although the CD spectra of IFNγR2 and IFNγR1 differ considerably (Černý *et al.*, 2015 ▸), both proteins belong to the same fold of the fibronectin type III domain family (Pfam PF00041).

Besides deglycosylation by the endoglycosidases, we designated asparagine-to-glutamine mutants to decrease the level of glycosylation. We prepared a fully mutated IFNγR2 variant bearing N56Q, N110Q, N137Q and N231Q mutations and single-point mutants N110Q, N137Q and N231Q, respectively. All of these constructs were transfected into insect S2 cells, but none of them were secreted into the cell-culture medium. This correlates with the earlier observation that IFNγR2 mutants with changed glycosylation patterns were located in the cytoplasmic fraction (Moncada-Vélez, 2013 ▸). Structurally significant is glycosylation at positions Asn110 and Asn137, where the bound *N*-acetyl-d-glucos­amine (NAG) residues sandwich Trp131 (Fig. 2 ▸ *b*), thus shielding its hydrophobic surfaces from solvent. As suggested by the failure to express and/or purify the N56Q, N110Q, N137Q and N231Q mutants, glycosylation is necessary for IFNγR2 production by stabilizing the fold and transport to and/or across the cellular membrane.

### 3.3. The IFNγR2 fold is stable without disulfide bonds   

IFNγR2 contains five cysteine residues, and our mass-spectrometric analysis identified disulfide bonds linking Cys86 to Cys94 and Cys209 to Cys234. We observed the same protein mobility under nonreducing and reducing conditions during SDS–PAGE analysis. The melting temperature of both glycosylated and deglycosylated IFNγR2 measured by thermal shift assay (Supplementary Fig. S2) decreased by only ∼1°C in the presence of 5 m*M* TCEP (a reducing agent to break the disulfide bonds), so that the IFNγR2 fold is stable without S—S bonds. This contrasts with the behaviour of IFNγR1 (Fountoulakis *et al.*, 1990 ▸), in which the protein fold is stabilized to a large extent by S—S bridges. The fifth IFNγR2 cysteine residue, Cys174, does not form an intramolecular S—S bridge but is bound to a monomeric cysteine. Binding of free cysteine to the sterically accessible Cys174 probably occurs after secreting IFNγR2 into the cell-culture medium, which contains free cysteine and stabilizes the monomeric form of IFNγR2.

### 3.4. The overall structure of the extracellular portion of IFNγR2   

The structure of IFNγR2 was solved at 1.8 Å resolution and electron density was observed for amino-acid residues 28–240 of UniProt entry P38484, except for two two-residue loops. Data-collection and refinement parameters are shown in Table 1 ▸. The extracellular part of the IFNγR2 molecule consists of two domains (Fig. 2 ▸), the N-terminal D1 domain of UniProt residues 28–133 and the C-terminal D2 domain of residues 144–247. Both domains belong to the immuno­globulin fold with fibronectin type III topology, forming β-sandwiches (Pfam PF00041). The inter-domain torsion angle is approximately 120°, similar to those of IFNγR1 (Thiel *et al.*, 2000 ▸; Walter *et al.*, 1995 ▸) and human tissue factor (Harlos *et al.*, 1994 ▸); the D1–D2 torsion angle is defined in Supplementary Table S3. The D1 domain is composed of three β-strands stacked on a layer of four β-strands, and the D2 domain is created by four β-strands arranged against four other β-strands; both domains are connected by a short linker (residues 134–143 in IFNγR2) comprising a short helix that is also found in IFNγR1, human tissue factor and other receptors.

### 3.5. Structural motifs in D1 and D2   

D1 contains a distinct structural motif of six stacked residues: Lys68, Trp74, Arg114, Trp126, Arg116 and His123. The average distance between the mean planes of the individual side chains of this extensive π–cation interaction is 3.65 Å. Analysis of the interaction energies in D1 revealed that the motif contributes significantly to the overall stability of the whole domain. These six surface residues are involved in interactions that are comparable in strength to the hydrophobic core of the domain and are likely to play an important role in the process of domain folding. The residues responsible for domain stability are depicted in Supplementary Fig. S3, in which the colour and thickness of the cartoon representation show the relative interaction energy per residue ranging from low (blue) to high (red) stabilizing values.

An analogous stacking motif with the consensus sequence WS*X*WS (Bazan, 1990 ▸) has been predicted by sequence alignments in D1 of the class 1 receptor family (McElroy *et al.*, 2009 ▸), but such a motif is missing in the D2 domains of both class 1 and class 2 receptors. Based on the presence of the KWRWRH motif in IFNγR2, we performed structural alignment of the class 2 receptor structures and discovered a similar but sequentially noncontinuous motif with the sequence (*X*)WRWR(*X*), where *X* is K, R or H. The important role of large aromatic tryptophan residues in stabilizing the fibronectin fold by stitching together two β-strands is accompanied in D1 by a structural role for charged residues, especially arginines. Besides the discussed (*X*)WRWR(*X*) motif, we found a tight overlap of a continuous chain of residues R-L/V-R-A (residues Arg114-Leu115-Arg116-Ala117 in IFNγR2): the average r.m.s.d. between motifs from two receptors is 0.6 Å. The other important residues that are conserved in D1 of the available class 2 receptor structures are residues corresponding to Trp49, Ser124 and the Cys86/Cys96 pair forming a disulfide bond in IFNγR2. A unique feature of IFNγR2 D1 is a short helix (residues 78–85), which is present in no other discussed receptor structure.

Sequential and structural comparison of D2 revealed a considerable sequence variability, within which we identified the conservation of two proline residues, Pro142 and Pro143, and the structurally well conserved motif 175-YNVA*X*W-180, with r.m.s.d. values about 1 Å but low sequence similarity. Another characteristic structural feature of D2 is the formation of a disulfide bridge between Cys209 and Cys234. Higher values for the *B* factors in D2 indicate its higher flexibility compared with D1. A higher flexibility of D2 compared with D1 was also indicated in our previous studies of IFNγR1 (Mikulecký *et al.*, 2013 ▸; Černý *et al.*, 2015 ▸).

### 3.6. Structural alignment of domains D1 and D2 in IFNγR2 and in other class 2 receptors   

We performed alignment of the IFNγR2 structure with the 11 remaining available structures of the class 2 receptor family in order to gauge their similarity and reveal their unique features. The alignment was measured by overlapping 34 residues in the N-terminal D1 and the same number of residues in the C-terminal D2; the r.m.s.d. values of the overlapped residues are listed in Fig. 3 ▸ and the overlapped residues are listed in Supplementary Table S2. The D1 domains are mutually more similar than the D2 domains, as highlighted by blue and red hues in Fig. 3 ▸; the average r.m.s.d. between two D1 domains is 0.95 Å and that between two D2 domains is 1.3 Å. A high similarity within D1 and D2, respectively, indicates that modulation of the specificity of receptors takes place in only a few variable regions, which are discussed below.

**Figure 4:**
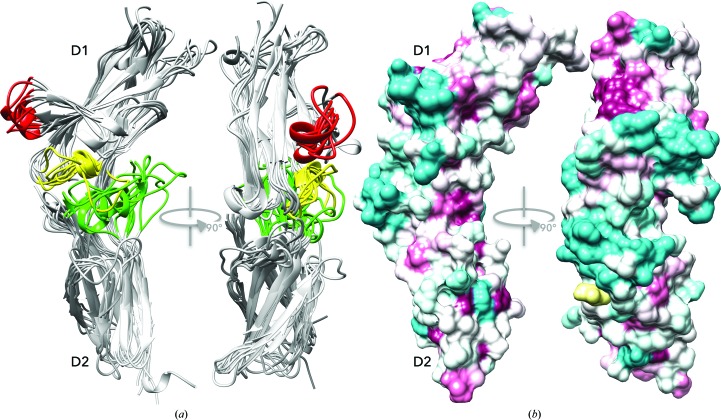
Structurally variable regions and a proposed interaction interface of the cytokine receptors. (*a*) Cartoon representation of the structural superposition of the D1 and D2 domains of the nine receptor structures. The backbone of the selected residues (listed in Supplementary Table S2) was superimposed independently for each domain and was then drawn on top of the IFNγR2 structure. The aligned cores of both domains overlap tightly (for r.m.s.d. values, see Fig. 3 ▸), but two regions in D1 (red and yellow) and one in D2 (green) are highly variable. (*b*) Sequences of IFNγR2 from 90 species with the sequentially most variable regions coloured cyan and conserved regions in purple drawn on the surface of the IFNγR2 structure by *ConSurf* (Glaser *et al.*, 2003 ▸). The IFNγR2 free cysteine Cys174 is highlighted in yellow. (*a*) and (*b*) show IFNγR2 in the same orientation.

D1 domains bear two conflicting structural features: strict fold conservation reflected by high structural similarity of the selected residues, and at the same time the presence of two structurally highly variable loops corresponding to residues 70–73 and 97–107 in IFNγR2. The third variable loop was located in the D2 domain (residues 162–171 in IFNγR2); the loops are coloured red and yellow for D1 and green for D2 in Fig. 4 ▸. Given the fairly uniform core of both domains and variability concentrated in the three localized regions, we suggest that the binding specificity of the individual receptors is controlled by these variable regions. However, there is another factor that contributes to the receptor specificity, the different mutual orientation of D1 and D2 (Supplementary Table S3), which displaces these variable regions to different positions, thus providing a unique binding interface for each receptor.

Several structural and sequential features of receptors of interferon-α and interferon-β (PDB entry 3se4; Thomas *et al.*, 2011 ▸), here labelled IFNαR1 and IFNαR2, distinguishes them from the other analyzed receptors. Specifically, IFNαR1 is composed of four instead of two domains; here, we analyzed D1 and D2. The D3–D4 pair cannot be analyzed as D4 is not resolved in the electron density. Further dissimilarities of IFNαR1 and IFNαR2 are found in the composition of their π–cation motifs: in IFNαR1 only four residues stack in D1 (Trp46, Arg76, Trp87 and Arg78) and three in D3 (Trp250, Arg279 and Trp291), while D1 of IFNαR2 does not have the motif at all and is replaced by the motif YVTV.

### 3.7. Similarity among receptors and consequences for evolution   

As discovered previously (Yoon *et al.*, 2010 ▸), an aromatic tyrosine or phenylalanine residue situated in the cleft between D1 and D2 of gp130 (PDB entry 1bqu; Bravo *et al.*, 1998 ▸), γ_c_ (PDB entry 4gs7; Ring *et al.*, 2012 ▸) and IL10R2 (PDB entry 3lqm; Yoon *et al.*, 2010 ▸) serves as the key binding epitope of promiscuous class 1 and 2 receptors, implying the existence of a common ancestor. Superposition of these receptor structures shows that the orientation of Phe109 and three residues in gp130, γ_c_ and IL10R2 are quite different (Fig. 2 ▸ *c*). No preferred rotamer of Phe109 overlaps the three former residues without significant rebuilding of the IFNγR2 backbone. The structural difference between IFNγR2 and the other receptors, especially IL10R2, is significant and suggests that there is not a common binding epitope for these receptors.

Significant sequence similarity between IFNγR2, IL10R2 and IL20R2 (20–25% sequence identity among different species; analysis not shown) points to their evolutionary relationship. If proven, it would be analogous to the evolutionary relationship between receptors 1 of the cytokines IFNγR1, IL10R1 and IL20R1 (Langer *et al.*, 2004 ▸). We may therefore hypothesize that these three cytokine systems have evolved from a common ancestral system: while interferon-γ evolved early in evolution and is known in fish species (Savan *et al.*, 2009 ▸), its receptor 2 emerged later in connection with the evolution of amphibians. The specific function of IFNγR2 therefore evolved from an older promiscuously functioning molecule. A likely candidate is IL10R2, because it is evolutionarily older and is known in primitive fishes, while IL20R2 emerges similarly to IFNγR2 in amphibians. The lack of a common binding epitope between IFNγR2 and the other class 2 receptors, notably IL10R2 (Fig. 2 ▸ *c*), indirectly supports this hypothesis.

### 3.8. The sequence alignment of IFNγR2 from various species suggests its binding interface   

In an attempt to identify the putative interface by which IFNγR2 forms a functional ternary complex with its binding partners interferon-γ and receptor 1, we aligned the IFNγR2 sequences from 90 species and used the *ConSurf* server to project the consensus onto the structure of IFNγR2 (Fig. 4 ▸ *b*). The 32 conserved residues (purple in Fig. 4 ▸ *b*) are mainly located in the inward arched part of the U-shaped receptor molecule. This part of the molecule contains the previously described stacking motif (Fig. 2 ▸ *a*) and plays an important role in maintaining the overall structure. The 34 most variable residues are predominantly on the opposite side of the molecule (cyan in Fig. 4 ▸ *b*). These regions of sequentially least conserved residues coincide with the location of the structurally variable loops derived by the superposition of receptor structures. We therefore conclude that the putative IFNγR2 interface for forming the active ternary complex with IFNγ and IFNγR1 is likely to be in the receptor 2 region with the most variable residues. This conclusion is supported by an analogous observation in the IFNγR1 system: the least sequentially conserved residues form the interface with the binding partner (Mikulecký *et al.*, 2013 ▸).

The composition of the ternary signalling complexes of dimeric cytokines discussed here, IFNγ and IL-10, is understood less than for monomeric examples such as IL-20, for which the ternary complex has a known crystal structure (Logsdon *et al.*, 2012 ▸). One of the reasons is the existence of two binding interfaces in the dimeric cytokines and the resulting different and more complex stoichiometry of the complexes; the crystal structure of an IFNγ–IFNγR1 complex with an unexpected 2:3 stoichiometry serves as an example (Thiel *et al.*, 2000 ▸). The topology and structure of the signalling ternary complex of IFNγ have been extensively studied and reviewed (Pestka *et al.*, 1997 ▸; Hoffmann *et al.*, 2015 ▸). Experiments in solution and on the cell surfaces indicated a 2:2:2 or 2:2:1 stoichiometry of the signalling IFNγ complex (Marsters *et al.*, 1995 ▸); cross-linking of different components of the IFNγ complex expressed in cloned cell lines have shown direct contact between IFNγR1 and IFNγR2 (Krause *et al.*, 2006 ▸) and also between IFNγ and IFNγR2 (Kotenko *et al.*, 1995 ▸). The newly determined structure of IFNγR2 may spur new experiments exploring the topology and three-dimensional structure of the signalizing ternary complex of IFNγ.

## 4. Conclusions   

A partially deglycosylated extracellular part of the interferon-γ receptor 2, IFNγR2, was crystallized and its structure was determined at 1.8 Å resolution and deposited in the PDB with accession code 5eh1. The electron-density map was interpreted for amino-acid residues 28–240, apart from two short loops. The IFNγR2 structure revealed the fold common to other cytokine receptors: two fibronectin type III domains connected by a short linker. IFNγR2 is a glycoprotein with five of the six potential N-linked glycosylation sites glycosyl­ated, as confirmed by mass spectrometry and the crystal structure. Our analysis of glycosylation also uncovers the role of the oligosaccharide moieties at Asn110 and Asn137, which sandwich Trp131 and shield its hydrophobic aromatic ring from the solvent. Both potential disulfide bonds form but are not critical for the stability of IFNγR2, as it is also stable in a reducing environment. The fifth cysteine Cys174 is bound to the monomeric cysteine.

Structure and sequence alignments revealed some important features of the 12 class 2 receptors. Their N-terminal D1 domains are more mutually similar than their C-terminal D2 domains (Fig. 3 ▸). D1 carries a distinctive so far unrecognized structural feature: a π–cation motif of sequentially distant stacked residues (*X*)WRWR(*X*) (KWRWRH in IFNγR2; Fig. 2 ▸ *a*). Analysis of the receptor structures revealed three structurally highly variable regions (Fig. 4 ▸ *a*), which most likely bring about binding specificity for their interacting partners. This hypothesis is further supported by the alignment of IFNγR2 sequences from various species, which identified the highest sequence variability at positions coinciding with the structurally variable regions (Fig. 4 ▸ *b*). An important structural feature distinguishing IFNγR2 from the related IL10R2, gp130 and γ_c_ receptors is the specific positioning of the aromatic recognition epitope in IFNγR2 (Fig. 2 ▸ *c*).

We believe that the determination of the structure of the so-far missing component of the interferon-γ signalling complex will enable a deeper understanding of the functioning of this important immunity cascade.

## 5. Related literature   

The following references are cited in the Supporting Information for this article: Cancino-Díaz *et al.* (2002 ▸), Černý *et al.* (2007 ▸), Furche *et al.* (2014 ▸), O’Boyle *et al.* (2011 ▸) and Rüger *et al.* (2015 ▸).

## Supplementary Material

PDB reference: interferon-γ receptor 2, 5eh1

Supporting Information.. DOI: 10.1107/S2059798316012237/dw5166sup1.pdf

## Figures and Tables

**Table 1:** Data-collection statistics and structure-refinement parameters Values in parentheses are for the highest resolution shell.

X-ray source	MX 14.1, HZB
Wavelength (Å)	0.91841
Total oscillation angle (°)	180
Resolution range (Å)	62.88–1.80 (1.91–1.80)
Space group	*P*6_1_22
Unit-cell parameters (Å)	*a* = *b* = 58.102, *c* = 377.266
No. of measured reflections	688675 (110540)
No. of unique reflections	36723 (5733)
Average multiplicity	18.8 (19.3)
Completeness (%)	99.9 (99.8)
Average *I*/σ(*I*)	17.6 (2.5)
Overall *B* factor from Wilson plot (Å^2^)	20
Average *B* factor (Å^2^)	28
No. of non-H atoms	
Protein	1734
Saccharides	42
Waters	311
All	2121
*R*_merge_	0.148 (1.395)
Half-data-set correlation coefficient CC_1/2_	99.9 (81.2)
No. of reflections, test set	1820
Final *R*_work_/*R*_free_/*R*_all_	0.190/0.222/0.191
Ramachandran plot	
Residues in favoured region	213 [96.3%]
Residues in allowed regions	219 [99.1%]
Outliers	2 [0.9%]
