# Supplementary material for: Crystal structure of human interferon-γ receptor 2 reveals the structural basis for receptor specificity
Source: Acta Crystallogr D Struct Biol. 2016 Aug 18;72(Pt 9):1017–25. doi: 10.1107/S2059798316012237 (PMC5013595; doi:10.1107/S2059798316012237)
Supplement: Supplementary file 1 [file d-72-01017-sup1.pdf]

STRUCTURAL BIOLOGY

Volume 72 (2016)

Supporting information for article:

Crystal structure of the human interferon gamma receptor 2 reveals the structural basis for receptor specificity

Pavel Mikulecký, Jirí Zahradník, Petr Kolenko, Jirí Cerný, Tatsiana Charnavets, Lucie Kolárová, Iva Necasová, Phuong Ngoc Pham and Bohdan Schneider

S1. Methods

S1.1. Cloning, expression, and purification of endoglycosidases. Endoglycosidases Endo H (residues 42-313; UniProt P04067) and PNGase F (residues 8-299; UniProt Q9XBM8; (Cancino-Diaz et al., 2002)) were produced in *Escherichia coli* BL21 (λDE3) and purified before deglycosylation trials. Briefly, genes were obtained as a GeneArt Strings DNA Fragments and cloned into previously modified vector pET26b(+) bearing StrepTag instead of 6x HisTag using *Nde*I and *Xho*I restriction enzymes. Cells were grown in 1 L of LB medium at 30 °C until the OD_{600} = 0.6 was reached, then the temperature was decreased to 16 °C and protein expression was induced by addition of 1 mM IPTG, and cells were incubated for another 20 hours. Two-step purification of enzymes consisted of gravity flow StrepTactin Sepharose followed by size exclusion chromatography on a HiLoad 16/600 Superdex 75 pg (GE Healthcare).

S1.2. Thermal shift assay (TSA). Melting temperature curves was also obtained from fluorescence-based Thermal shift assay using fluoroprobe. Experiment was performed in "CFX96 Touch Real-Time PCR Detection System" (Bio-Rad) using FRET Scan Mode. The concentration of fluorescent SYPRO Orange dye (Sigma Aldrich) was 8-fold dilution from 5,000-fold stock and protein concentration was 0.05 mg/mL in final volume of 25 µL. As a reference was used only "HN Buffer" without protein. Thermal denaturation of proteins was performed in capped "Low Tube Strips, CLR" (Bio-Rad) and possible air bubbles in samples were removed by centrifugation immediately before the assay. The samples were heated from 20 to 95 °C with stepwise increment of 0.5 °C/min and a 30 s hold step for every point, followed by the fluorescence reading. Data (after subtraction of reference sample) were normalized and used for first derivative calculation to estimate the melting temperature.

Table S1. Primers for mutagenesis. The IFNgR2 variants bearing single mutations: N110Q, N137Q, and N231Q were produced using the QuikChange II Site-Directed Mutagenesis Kit (Agilent Technologies). Primer pair (forward primer – Fwd and reverse – Rev) are marked by mutation code.

Primer name	Sequence (mutant position highlighted)
FwdN137Q:	5'-GTTTCAACACTATCGGCAAGTGACTGTCGGGCCTC-3'
RevN137Q:	5'-GAGGCCCGACAGTCACTTGCCGATAGTGTTGAAAC-3'
FwdN110Q:	5'-CTTCCCAATGGATTTCCAGGTCACTCTACGCCTTCG-3'
RevN110Q:	5'-CGAAGGCGTAGAGTGACCTGGAAATCCATTGGGAAG-3'
FwdN231Q:	5'-GAGTCGGGCATTTAAGCCAGATATCTTGCTACGAAACAATG-3'
RevN231Q:	5'-CATTGTTTCGTAGCAAGATATCTGGCTTAAATGCCCGACTC-3'

Table S3. The mutual orientation of the D1 and D2 domains of the cytokine receptors measured as torsion angles. The torsion angles are defined by CA atoms of four residues highly conserved among all receptors.

PDB and chain IDs	Receptor name	Torsion	Residues defining torsion between D1 and D2			
			Residue 1	Residue 2	Residue 3	Residue 4
5eh1_A	IFNgR2	-38º	A92	R89	Y150	V152
3lqm_A	IL10R2	-49º	A91	R88	Y154	V156
1fg9_C	IFNgR1	-30º	A87	R84	Y155	V157
1j7v_R	IL10R1	-14º	A81	R78	Y146	I148
4doh_B	IL20R2	-82º	A115	R112	F165	V167
4doh_E	IL20R1	-27º	A114	R111	Y181	V183
3dlq_R	IL22R	-21º	A98	R95	Y165	L167
3g9v_A	IL22BP	-40º	A105	R102	Y171	V173
3og6_B	IL28R	-17º	T82	R79	Y138	V140

Table S2. Amino acid residues (numbered as in the PDB structures) used to calculate root mean square deviations (rmsd, [Å]) for the D1 and D2 domains of the twelve analyzed receptor molecules listed in Figure 3. Note that the number of superimposed residues is the same in all domains, 34.

Calculation of rmsd of 34 residues of the N-terminal D1 domain

PDB ID	chain ID	receptor	residue number as numbered in the PDB file																																	
1fg9	C	IFNgR1	28	29	30	31	41	42	43	44	45	46	47	56	57	58	59	60	65	66	67	68	69	81	82	83	84	85	86	87	88	94	96	97	98	99
5eh1	A	IFNgR2	21	22	23	24	37	38	39	40	41	42	43	49	50	51	52	61	66	67	68	69	70	86	87	88	89	90	91	92	93	99	101	102	103	104
1j7v	R	IL10R1	21	22	23	24	35	36	37	38	39	40	41	48	49	50	51	52	57	58	59	60	61	75	76	77	78	79	80	81	82	88	90	91	92	93
3lqm	A	IL10R2	37	38	39	40	51	52	53	54	55	56	57	62	63	64	65	66	71	72	73	74	75	85	86	87	88	89	90	91	92	98	100	101	102	103
4doh	B	IL20R2	51	52	53	54	65	66	67	68	69	70	71	83	84	85	86	89	94	95	96	97	98	109	110	111	112	113	114	115	116	122	124	125	126	127
4doh	E	IL20R1	53	54	55	56	68	69	70	71	72	73	74	81	82	83	84	87	92	93	94	95	96	108	109	110	111	112	113	114	115	121	123	124	125	126
3dlq	R	IL22R	38	39	40	41	52	53	54	55	56	57	58	65	66	67	68	71	76	77	78	79	80	92	93	94	95	96	97	98	99	106	107	108	109	110
3g9v	A	IL22BP	44	45	46	47	59	60	61	62	63	64	65	72	73	74	75	78	83	84	85	86	87	99	100	101	102	103	104	105	106	112	114	115	116	117
3og6	B	IL28R	20	21	22	23	34	35	36	37	38	39	40	48	49	50	51	54	59	60	61	62	63	76	77	78	79	80	81	82	83	89	91	92	93	94
3se4	A	IFNabR1	17	18	19	20	33	34	35	36	37	38	39	46	47	48	49	52	57	58	59	60	61	73	74	75	76	77	78	79	80	86	87	88	89	90
3se4	C	IFNabR2	24	25	26	27	38	39	40	41	42	43	44	52	53	54	55	58	63	64	65	66	67	79	80	81	82	83	84	85	86	93	94	95	96	97
2puq	T	Tissue_F	22	23	24	25	33	34	35	36	37	38	39	45	46	47	48	49	54	55	56	57	58	71	72	73	74	75	76	77	78	93	94	95	96	97

Calculation of rmsd of 34 residues of the C-terminal D2 domain

PDB ID	chain ID	receptor	residue number as numbered in the PDB file																																	
1fg9	C	IFNgR1	113	114	115	116	117	118	124	125	126	127	128	129	152	154	155	156	157	158	159	184	185	186	187	197	198	199	200	201	202	203	204	213	214	216
5eh1	A	IFNgR2	118	120	121	122	123	124	130	131	132	133	134	135	148	149	150	151	152	153	154	171	172	173	174	184	185	186	187	188	189	190	191	203	204	206
1j7v	R	IL10R1	106	108	109	110	111	112	118	119	120	121	122	123	144	145	146	147	148	149	150	168	169	170	171	181	182	183	184	185	186	187	188	197	198	200
3lqm	A	IL10R2	116	117	118	119	120	121	127	128	129	130	131	132	152	153	154	155	156	157	158	175	176	177	178	188	189	190	191	192	193	194	195	204	205	207
4doh	B	IL20R2	141	142	143	144	145	146	152	153	154	155	156	157	163	164	165	166	167	168	169	189	190	191	192	202	203	204	205	206	207	208	209	218	219	221
4doh	E	IL20R1	140	141	142	143	144	145	151	152	153	154	155	156	179	180	181	182	183	184	185	202	203	204	205	215	216	217	218	219	220	221	222	232	233	234
3dlq	R	IL22R	124	125	126	127	128	129	135	136	137	138	139	140	163	164	165	166	167	168	169	185	186	187	188	198	199	200	201	202	203	204	205	214	215	216
3g9v	A	IL22BP	131	132	133	134	135	136	142	143	144	145	146	147	169	170	171	172	173	174	175	192	193	194	195	206	207	208	209	210	211	212	213	222	223	225
3og6	B	IL28R	108	109	110	111	112	113	119	120	121	122	123	124	136	137	138	139	140	141	142	161	162	163	164	175	176	177	178	179	180	181	182	192	193	195
3se4	A	IFNabR1	104	105	106	107	108	109	115	116	117	118	119	120	136	137	138	139	140	141	142	159	160	161	162	172	173	174	175	176	177	178	179	188	189	191
3se4	C	IFNabR2	110	111	112	113	114	115	121	122	123	124	125	126	139	140	141	142	143	144	145	167	168	169	170	180	181	182	183	184	185	186	187	195	196	198
2puq	T	Tissue_F	111	112	113	115	116	117	123	124	125	126	127	128	151	152	153	154	155	156	157	174	175	176	177	186	187	188	189	190	191	192	193	204	205	207

Calculation of rmsd of 34 residues providing the best overlap between D1 and D2 within each receptor molecule

PDB ID	chain ID	receptor	residue number as numbered in the PDB file																																	
1fg9	C	IFNgR1N	15	18	19	20	24	26	27	30	40	42	43	44	45	46	47	48	64	66	67	68	80	81	82	84	85	86	87	88	89	91	92	93	102	103
1fg9	C	IFNgR1C	113	115	116	117	121	123	124	127	152	155	156	157	158	159	160	161	172	183	184	185	195	196	197	199	200	201	202	203	204	208	209	210	219	220
5eh1	A	IFNgR2N	8	9	10	11	12	13	14	15	21	22	23	24	38	39	40	41	42	45	64	65	66	67	68	69	81	87	88	89	90	91	93	97	101	102
5eh1	A	IFNgR2C	141	142	143	144	145	149	151	152	153	155	168	169	170	171	172	173	174	175	177	178	179	180	181	182	183	184	185	195	196	198	199	200	201	202
1j7v	R	IL10R1N	7	8	9	10	11	13	20	21	24	25	32	39	40	41	45	46	47	50	57	58	59	60	61	62	67	74	76	77	78	79	81	82	89	90
1j7v	R	IL10R1C	105	106	107	108	109	111	118	119	122	123	142	149	150	151	156	157	158	161	166	167	168	169	170	171	174	179	181	182	183	184	186	187	196	197
3lqm	A	IL10R2N	27	29	37	48	50	51	52	53	54	56	60	63	64	71	72	73	74	75	76	86	87	88	89	90	91	92	93	95	96	103	104	105	106	107
3lqm	A	IL10R2C	118	120	128	149	152	153	154	155	156	158	164	167	168	173	174	175	176	177	178	188	189	190	191	192	193	194	195	199	200	208	209	210	211	212
4doh	B	IL20R1N	63	66	67	68	69	70	71	72	73	74	75	77	79	88	89	102	104	106	107	108	109	110	111	112	113	114	115	119	121	122	123	124	125	126
4doh	B	IL20R1C	171	174	176	178	179	180	181	182	183	184	185	186	191	193	194	195	196	197	198	210	213	214	215	216	217	218	219	220	221	222	224	226	234	235
4doh	E	IL20R2N	38	52	64	65	66	67	68	69	70	71	72	98	99	108	109	110	111	112	113	114	115	116	117	118	119	120	121	122	123	124	125	130	131	132
4doh	E	IL20R2C	141	154	163	164	165	166	167	168	169	170	171	191	192	200	201	202	203	204	205	206	207	208	209	210	213	214	215	216	217	218	219	223	224	225
3dlq	R	IL22RN	20	22	25	26	27	28	29	30	39	41	43	44	45	46	47	48	51	52	53	54	55	56	57	58	59	62	90	98	100	104	105	106	107	112
3dlq	R	IL22RC	121	122	123	124	125	126	134	164	165	166	167	168	169	170	171	198	200	202	203	204	210	211	212	213	214	215	216	217	218	219	220	221	222	224
3g9v	A	IL22BPN	30	31	34	35	36	43	44	45	46	48	49	50	58	59	60	61	62	63	64	75	81	82	83	100	101	102	103	104	105	111	112	113	114	115
3g9v	A	IL22BPC	130	131	133	134	135	142	143	144	145	147	148	149	169	170	171	172	173	174	175	186	189	190	191	206	207	208	209	210	211	219	220	221	222	223
3og6	B	IL28RN	7	17	19	20	21	24	31	32	34	35	36	37	38	39	40	46	58	62	63	64	65	74	76	79	80	81	82	83	87	88	89	90	91	92
3og6	B	IL28RC	108	116	119	120	121	124	134	135	137	138	139	140	141	142	143	147	157	162	163	164	165	172	174	177	178	179	180	181	188	189	190	191	192	193
3se4	A	IFNabR1N	11	13	18	19	20	33	34	35	36	37	38	39	40	41	47	48	54	58	59	60	71	72	73	74	75	76	77	78	79	80	87	88	93	94
3se4	A	IFNabR1C	107	109	117	118	119	137	138	139	140	141	142	143	144	145	151	152	155	158	159	160	169	170	171	172	173	174	175	176	177	178	188	189	194	195
3se4	C	IFNabR2N	39	40	41	42	43	50	52	53	54	59	66	67	68	69	70	72	73	74	77	78	79	80	81	82	83	84	85	94	95	96	97	98	99	100
3se4	C	IFNabR2C	141	142	143	144	145	149	151	152	153	155	168	169	170	171	172	173	174	175	177	178	179	180	181	182	183	184	185	195	196	198	199	200	201	202
2puq	T	TissueFN	6	13	14	22	23	24	25	26	35	36	39	40	41	44	45	46	47	54	55	56	57	58	59	70	71	72	73	74	75	76	93	94	99	100
2puq	T	TissueFC	108	115	116	124	125	126	127	128	154	155	158	159	160	164	165	166	167	172	173	174	175	176	177	184	185	186	187	188	189	190	203	204	209	210

Figure S1. SDS-PAGE analysis of the purified glycosylated and deglycosylated IFNgR2. The 12.5% Tris-Glycine gel was stained using Coomassie Brilliant Blue. Lane M – molecular-weight markers with depicted mass in kDa; Lane R2g – glycosylated IFNgR2; Lane R2d – IFNgR2 deglycosylated by Endo H endoglycosidase.

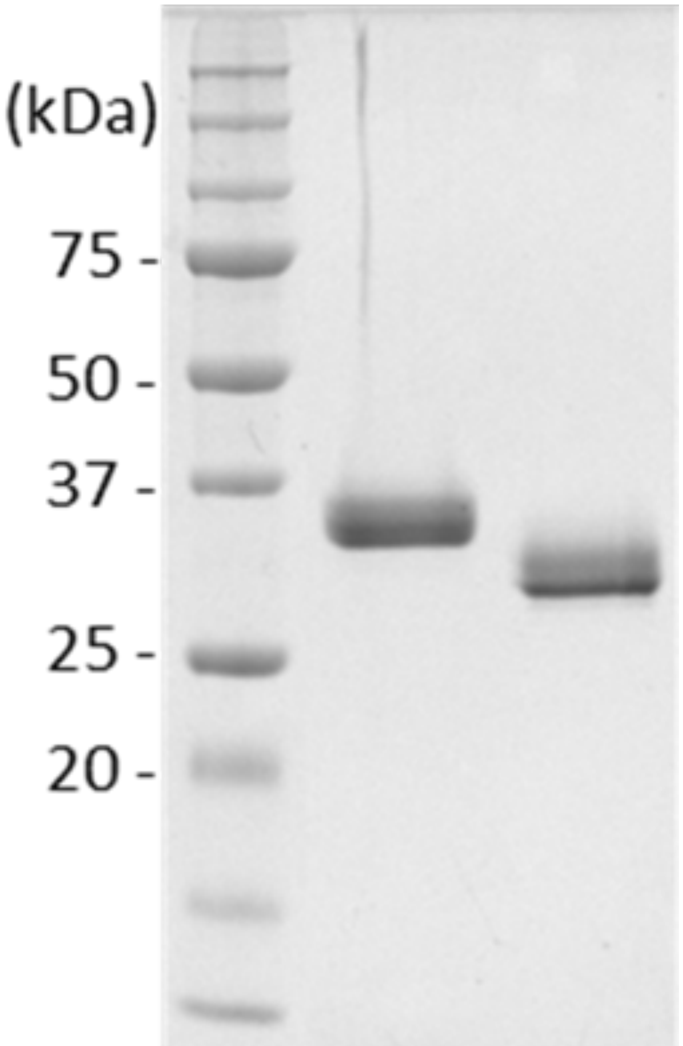

Figure S2. Normalized melting curves measured by thermal shift assay (TSA). Data showed that melting temperature of deglycosylated IFNgR2 (48 °C) is slightly lower than of glycosylated variant (50.5 °C). The addition of 5 mM TCEP to break the disulfide bonds lower the melting temperature just by one degree of Celsius of both deglycosylated and glycosylated IFNgR2 (47 and 49.5 °C, respectively).

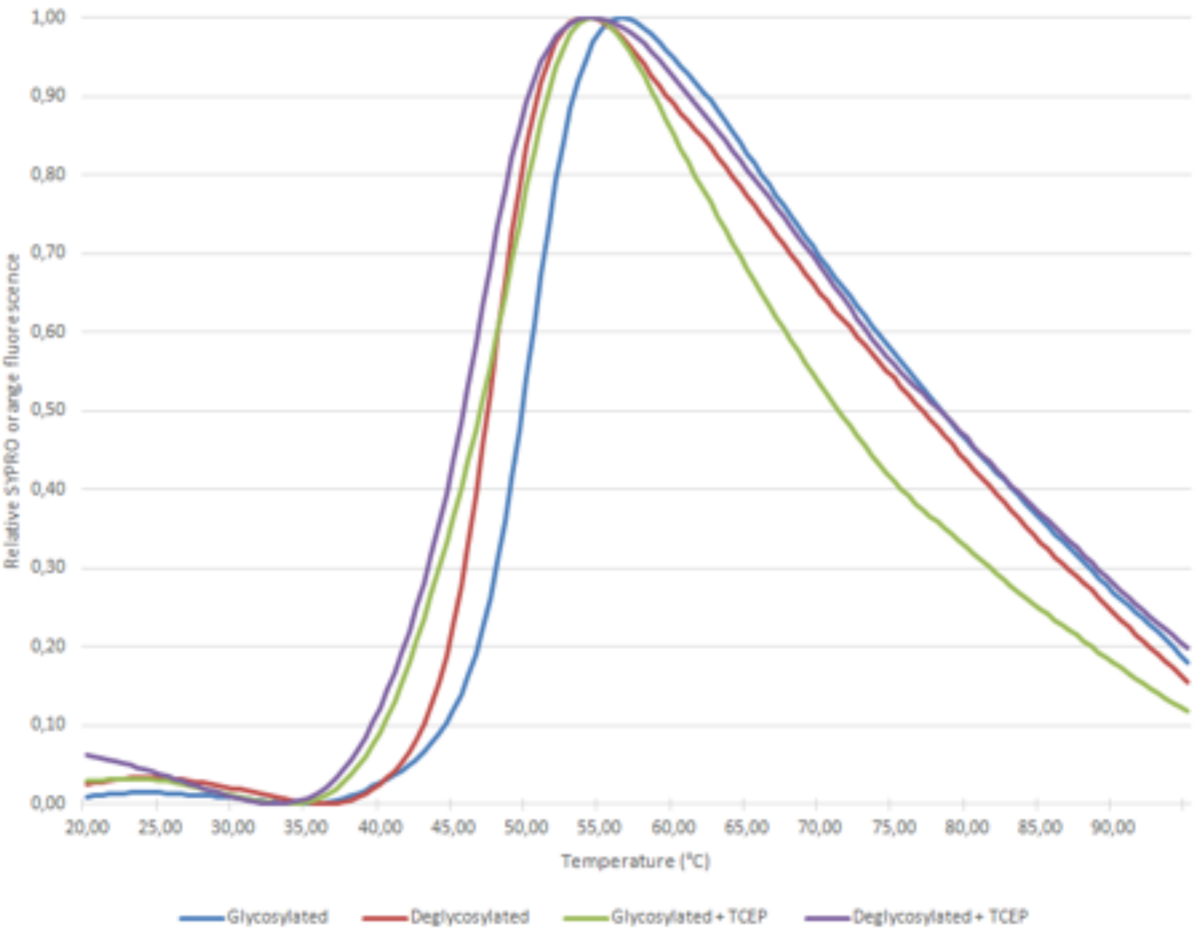

Figure S3. Residues responsible for stability of the IFNgR2 N-terminal (D1) domain. The color and thickness of the cartoon representation show relative interaction energy per residue ranging from low (blue) to high stabilizing values (red). The interaction energy within D1 was calculated at the DFT-D level (RI-TPSS/TZVP augmented with empirical dispersion term (Černý et al., 2007)) with the solvation effects described using COSMO in program TurboMole (Furche et al., 2014). All pairs of D1 residues within 4 Å were extracted from the 5eh1 structure using VMD; it was 113 residues forming 580 pairs. Hydrogen atoms were added using OpenBabel (O'Boyle et al., 2011) and their positions were optimized at the DFTB-D level of theory as implemented in dftb+ program (Rüger et al., 2015). Pairwise interaction energy was calculated at the DFT-D level and contributions involving each residue were summed.

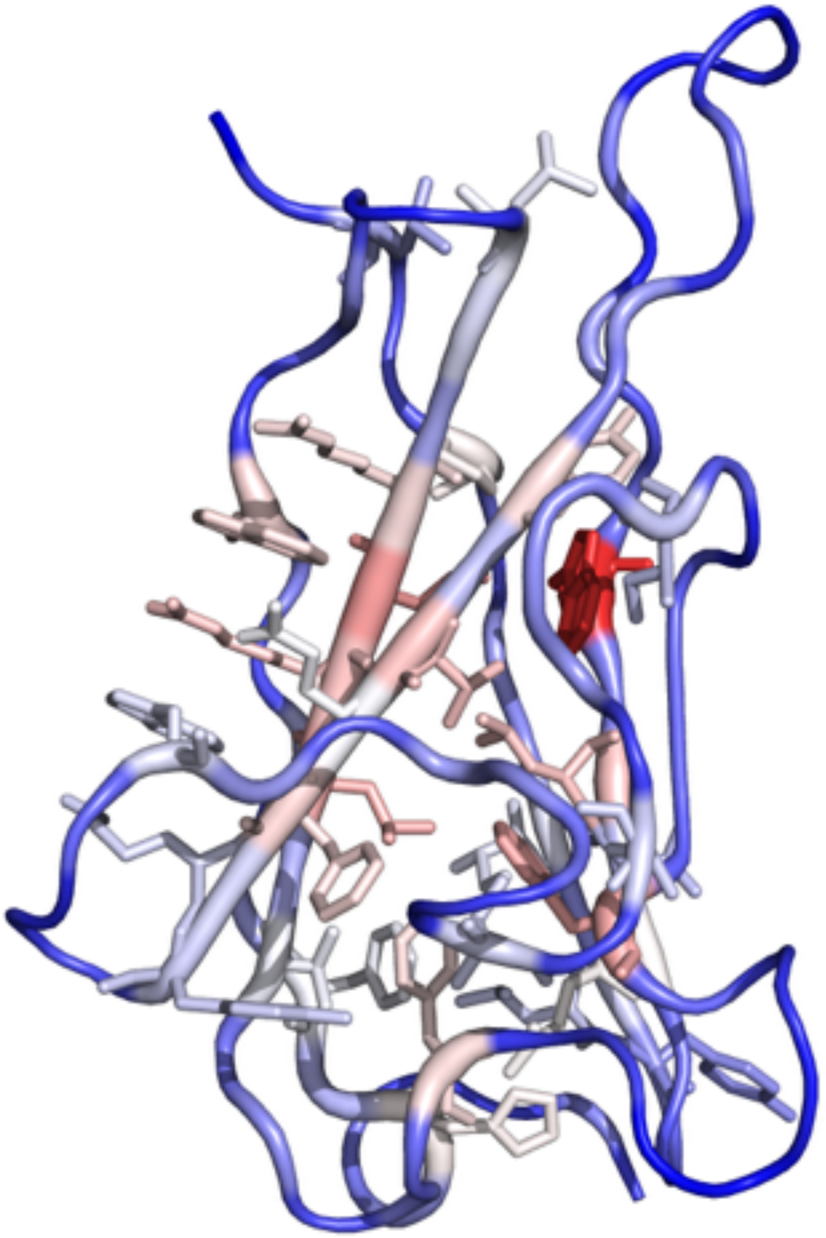

Bibliography

Cancino-Diaz, J. C., Reyes-Maldonado, E., Banuelos-Panuco, C. A., Jimenez-Zamudio, L., Garcia-Latorre, E., Leon-Dorantes, G., Blancas-Gonzalez, F., Paredes-Cabrera, G. & Cancino-Diaz, M. E. (2002). *J. Investig. Dermatol.* **119**, 1114-1120.

Černý, J., Jurečka, P., Hobza, P. & Valdés, H. (2007). *The Journal of Physical Chemistry A* **111**, 1146-1154.

Furche, F., Ahlrichs, R., Hättig, C., Klopper, W., Sierka, M. & Weigend, F. (2014). *Wiley Interdisciplinary Reviews: Computational Molecular Science* **4**, 91-100.

O'Boyle, N. M., Banck, M., James, C. A., Morley, C., Vandermeersch, T. & Hutchison, G. R. (2011). *Journal of Cheminformatics* **3**, 33-33.

Rüger, R., van Lenthe, E., Lu, Y., Frenzel, J., Heine, T. & Visscher, L. (2015). *Journal of Chemical Theory and Computation* **11**, 157-167.
